# Prognostic factors and clinical survival outcome in patients with primary mediastinal diffuse large B-cell lymphoma in rituximab era: A population-based study

**DOI:** 10.1097/MD.0000000000037238

**Published:** 2024-02-23

**Authors:** Haifang Hang, Hui Zhou, Liyuan Ma

**Affiliations:** a Department of Hematology, Shanghai Ninth People’s Hospital Affiliated to Shanghai Jiaotong University School of Medicine, Shanghai, China; b Department of Nursing department, Shanghai Ninth People’s Hospital Affiliated to Shanghai Jiaotong University School of Medicine, Shanghai, China.

**Keywords:** Nomogram, primary mediastinal diffuse large B cell lymphoma, prognosis, SEER database

## Abstract

The goal of this study was to investigate the clinical characteristics, prognostic variables, and survival of patients with primary mediastinal diffuse large B cell lymphoma (PMBCL) in the rituximab era. The Surveillance, Epidemiology, and End Results (SEER) database was used to identify PMBCL patients diagnosed between 2000 and 2019. The Kaplan–Meier (K-M) technique and log-rank test were used to assess overall survival (OS) and disease-specific survival (DSS). The independent prognostic variables for OS and DSS were identified using univariate and multivariate Cox regression analysis. Nomograms were created to predict survival prospects according to identified prognostic indicators. Totally, 841 patients were enrolled with PMBCL. One-year, 5-year, and 10-year OS rates were 93.99%, 85.04%, and 81.76%, and the corresponding DSS rates were 95.27%, 87.37%, and 85.98%. The results of multivariate Cox regression analysis demonstrated that age, years of diagnosis, Ann arbor staging, and chemotherapy were independent prognostic factors for survival. Nomograms designed exclusively for PMBCL were created to forecast the likelihood of 1-year, 5-year, and 10-year OS and DSS, respectively. The Harrell concordance index (C-index) for the nomograms predictions of OS and DSS were 0.704 and 0.733, respectively, which showed the established model harboring powerful and accurate performance. The present study revealed that incidence of PMBCL has been consistently rising over the last 20 years. Simultaneously, survival rates have improved tremendously. Rituximab based immunochemotherapy has emerged as an effective treatment option, leading to enhanced OS and DSS outcomes. Furthermore, the nomograms specifically developed for PMBCL have demonstrated robustness and accuracy in forecasting OS and DSS rates at 1, 5, and 10 years. These predictive tools can be valuable for clinicians in accurately estimating prognosis and establishing personalized treatment plans and follow-up protocols.

## 1. Introduction

Primary mediastinal large B-cell lymphoma (PMBCL) constitutes a minority proportion, ranging from 2% to 4%, of all non-Hodgkin lymphoma. This particular type of lymphoma predominantly affects individuals in their youth (median age of 35 years old), and exhibits a higher incidence among females.^[1–4]^ Patients may encounter clinical onset caused by the tumor compression or superior vena cava syndrome (SVCS). The majority of patients are diagnosed with Ann Arbor stage I or II disease, however there is a potential of extension into the adjacent organs(lung, chest wall, pleura, pericardium). In 10% of cases, extrathoracic disease may develop including adrenal gland, intracerebral, and kidney infiltration.^[3,5]^ According to the Revised European American Lymphoma Classification, PMBCL was previously categorized as a subtype of diffuse large B-cell lymphoma due to its unique clinicopathologic characteristics. Nevertheless, molecular biological research has revealed a distinct gene expression pattern (GEP) in PMBCL that closely resembles classic Hodgkin lymphoma (cHL). As a result, PMBCL has been recognized as a separate entity in the World Health Organization (WHO) classification.^[6–8]^ Recurrent molecular abnormalities such as 9p24.1 and 2p16.1 leading to the constitutive activation of the JAK-STAT or NF-kB pathways, were widely acknowledged as significant characteristics of the PMBCL disease.^[9–12]^ Moreover, there is a prevailing immune evasion phenotype that is accomplished through a multitude of genetic mechanisms, including modifications in the genetic structure at 9p24.1, resulting in the expression of PDL1 and, notably, PDL2.^[13,14]^ In addition to advancements in comprehending the biological aspects of PMBCL, numerous clinical trials and real world studies have proven enhanced outcomes through the use of rituximab based immunochemotherapy. However, there is ongoing debate regarding the optimal initial treatment and the position of consolidation radiotherapy (RT). Moreover, there is a strong scientific underlying mechanism for inhibiting the PD1-PDL pathway, leading to investigations of immune checkpoint inhibitors in relapsed/refractory (R/R) PMBCL cohort, demonstrated remarkable efficacy and have resulted in the approval of these medications for this particular situation.^[15]^

Nevertheless, our current recognition of PMBCL is predominantly built upon small-scale case reports or retrospective studies. There have been few extensive studies conducted on the incidence, treatment, and survival rates of PMBCL on a large scale. Fortunately, the Surveillance, Epidemiology, and End Results (SEER) contributes invaluable resources in exploring rare malignant diseases such as PMBCL in areas where retrospective or prospective clinical trials data are unavailable. Thus far, this SEER database study stands as the most extensive and up-to-date PMBCL cohort documented. Thereby, we exploited the SEER to primarily delve into prognostic variables and survival condition of PMBCL in the early twenty-first century. Additionally, we endeavored to identify independent prognostic markers associated with PMBCL and construct nomograms facilitating clinicians in accurately predicting prognosis.

## 2. Materials and methods

### 2.1. Patients

The SEER database is a comprehensive and representative tumor registration database in North America. Data was collected using the SEER Stat software (Version 8.4.1) from SEER Research Plus, 17 Registries (2000–2019, Nov2021 Sub) database, which was released in March 2023. The third version of the International Classification of Diseases for Oncology (ICD-O-3) was used to identify the PMBCL subtype,specifically the Site and Morphology-Historic Lymphoma subtype record/WHO 2008, 2(a) 2.3.4 mediastinal large B cell lymphoma. Various demographic and clinicopathological information such as age, gender, race, marital condition, Ann Arbor staging, B symptom presentation, surgery performance, radiation performance, chemotherapy acceptance, years of diagnosis, survival status, survival time, and cause of death were obtained. Patients with a prior cancer diagnosis and those without microscopic confirmation were excluded from the study. Patients with missing demographic, clinicopathological, and follow-up information were also excluded. The PMBCL patients were divided into 2 groups based on the year of diagnosis (2000–2010 and 2011–2019) to analyze the trends in survival over the last 2 decades. Additionally, the patients were divided into 2 age groups (under 60 and over 60) to explore the impact of age on PMBCL survival. The main clinical outcome endpoints of the study including overall survival (OS) and disease-specific survival (DSS).

### 2.2. Statistical analyses

The estimated OS and DSS were calculated using Kaplan–Meier (K-M) technique and compared using the log-rank test. Both univariate and multivariate analyses utilized the Cox regression model. Independent factors that showed a significant difference in the univariate Cox regression analysis were further examined in the multivariate Cox regression analysis. Hazard ratios (HRs) and 95% confidence intervals (CIs) were calculated in Cox regression analysis, with the HRs being exponentiated to determine the effect/CI mode. Results of Cox regression were presented using Frost plots, which employed meta-analysis. The pooled Cox regression analysis results in PMBCL patients were used to depict nomograms for forecasting 1-year, 5-year, and 10-year OS and DSS. The concordance probability of the nomograms was estimated by Harrell concordance index (C-index). All statistical analyses were performed using STATA (version 12.0) software. Statistical significance was defined as a 2-sided *P* value <.05.

## 3. Results

### 3.1. Demographics characteristics of PMBCL patients

From 2000 to 2019, totally 841 patients with PMBCL were enrolled in the SEER Research Plus, 17 Registries (2000–2019, Nov2021 Sub) database. The cohort was composed by 331 (39.3%) males and 510 (60.7%) females patients. Vast majority of patients (92.0%) were younger than 60 years old, and white ethnicity (76%) was predominance. All patients were diagnosed between 2000 and 2019, with 324 (38.5%) diagnosed between 2000 and 2010, and 517 (61.5%) diagnosed between 2011 and 2019. Marital status was categorized as married (47.2%), unmarried (39.7%), divorced (5.0%), and widowed (2.0%). The Ann Arbor lymphoma clinical stage system was obtained from the database, with stage I accounting for 25.4% of cases, stage II for 35%, stage III for 6.5%, stage IV for 9.6%, and an unknown stage for 23.5% of cases. B symptoms, which are systemic symptoms associated with lymphoma, were present in 201 patients (23.9%), while 310 patients (36.9%) did not exhibit these symptoms. The majority of PMBCL patients (93.6%) received chemotherapy, while only approximately one-third received radiation therapy. Surgical intervention was performed in 97 (11.5%) patients. Table 1 demonstrated comprehensive clinical characteristics of these PMBCL patients.

**Table 1:** Patient characteristics of primary mediastinal diffuse large B-cell lymphoma diagnosed in SEER 17 registries, 2000 to 2019.

Characteristic	No. of patients	Percentage (%)
Total	841	100
Age at diagnosis		
60	774	92
≥60	67	8
Sex		
Male	331	39.3
Female	510	60.7
Race		
White	639	76
Black	87	10
Asian or pacific islander	97	12
American Indian/Alaska native	5	0.5
Unknown	13	1.5
Years of diagnosis		
2000–2010	324	38.5
2011–2019	517	61.5
Marital status		
Married	397	47.2
Unmarried	334	39.7
Divorced	42	5
Widow	17	2
Unknown	51	6.1
Ann Arbor stage		
I	213	25.4
II	294	35
III	55	6.5
IV	81	9.6
Unknown	198	23.5
B symptoms		
Presented	201	23.9
No	310	36.9
Unknown	330	39.2
Chemotherapy		
Performed	787	93.6
No	54	6.4
Radiation		
Performed	294	35
No	525	62.4
Unknown	22	2.6
Surgery		
Performed	97	11.5
No	736	87.5
Unknown	8	1

### 3.2. Survival analysis

**Figure 1.**
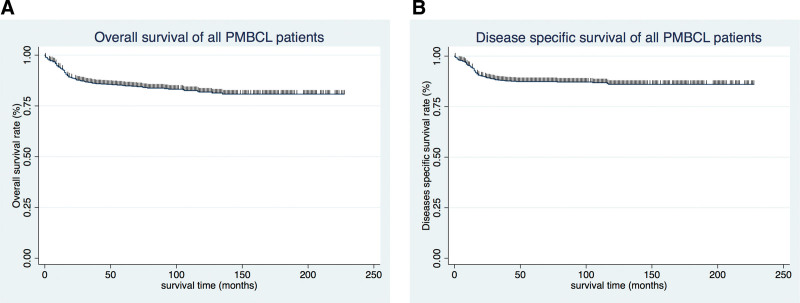
Survival analysis of primary mediastinal large B-cell lymphoma: (A) OS and (B) DSS were shown for all patients. DSS = disease-specific survival, OS = overall survival.

**Figure 2.**
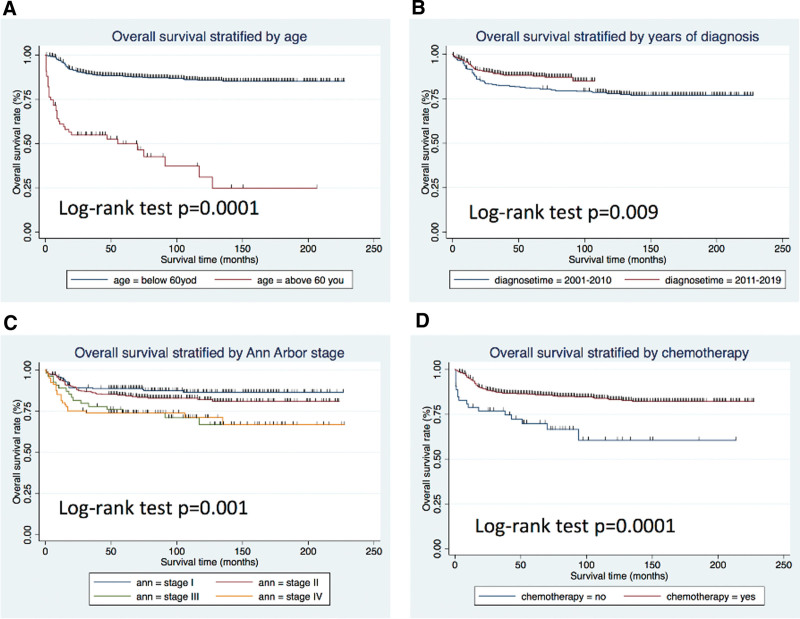
Overall survival analysis of primary mediastinal large B-cell lymphoma stratified by: (A) age, (B) years of diagnosis, (C) Ann Arbor stage, and (D) chemotherapy.

**Figure 3.**
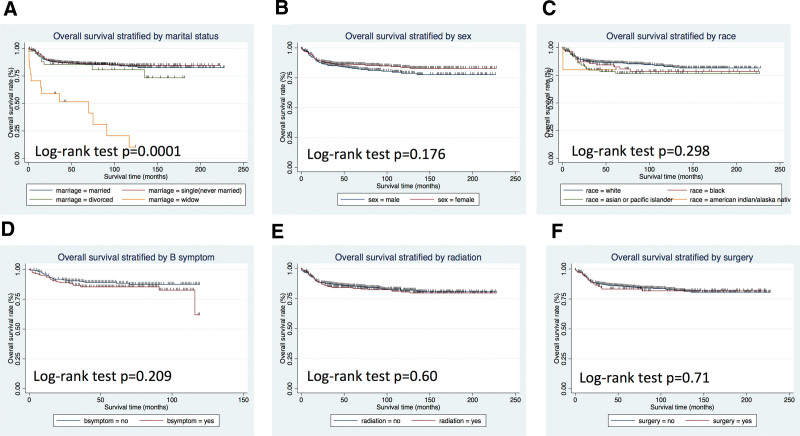
Overall survival analysis of primary mediastinal large B-cell lymphoma stratified by: (A) marital status, (B) sex, (C) race, (D) B symptoms, (E) radiation, and (F) surgery.

**Figure 4.**
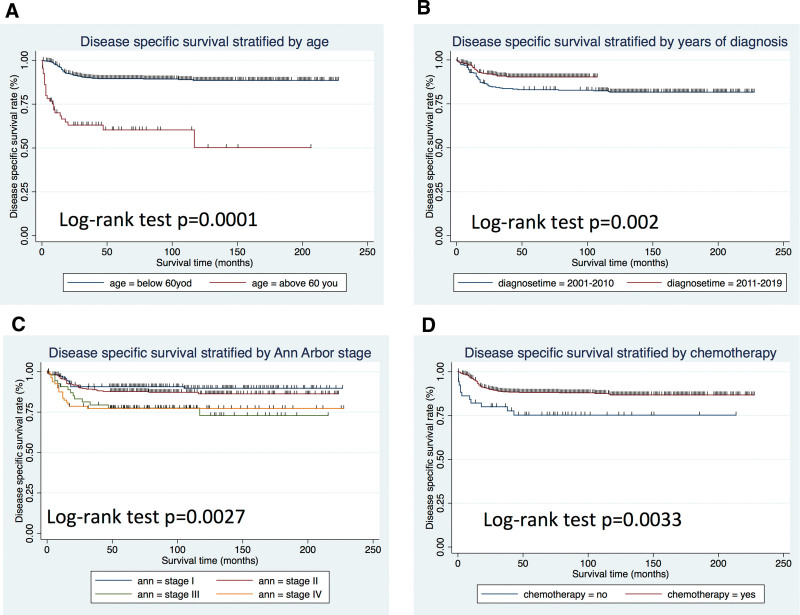
Diseases specific survival analysis of primary mediastinal large B-cell lymphoma stratified by: (A) age, (B) yr of diagnosis, (C) Ann Arbor stage, and (D) chemotherapy.

**Figure 5.**
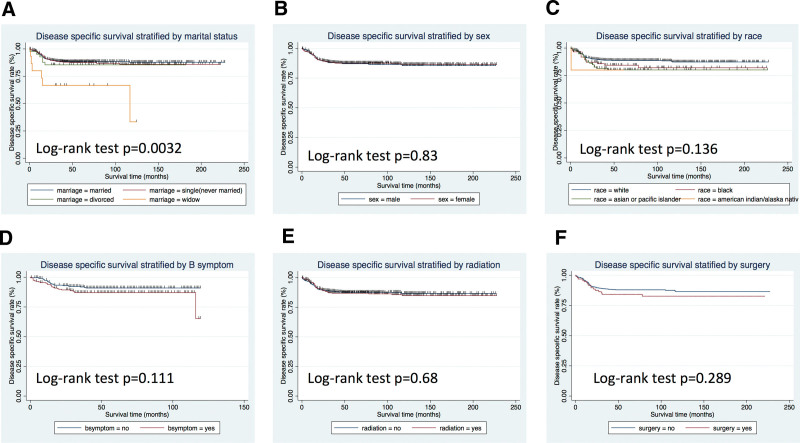
Diseases specific survival analysis of primary mediastinal large B-cell lymphoma stratified by: (A) marital status, (B) sex, (C) race, (D) B symptoms, (E) radiation, and (F) surgery.

**Figure 6.**
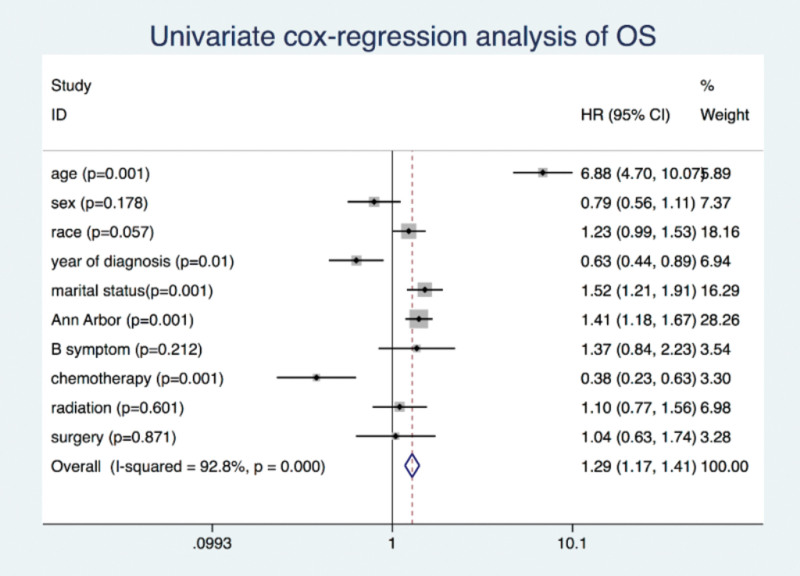
Univariate cox regression analysis of overall survival in primary mediastinal large B-cell lymphoma patients.

**Figure 7.**
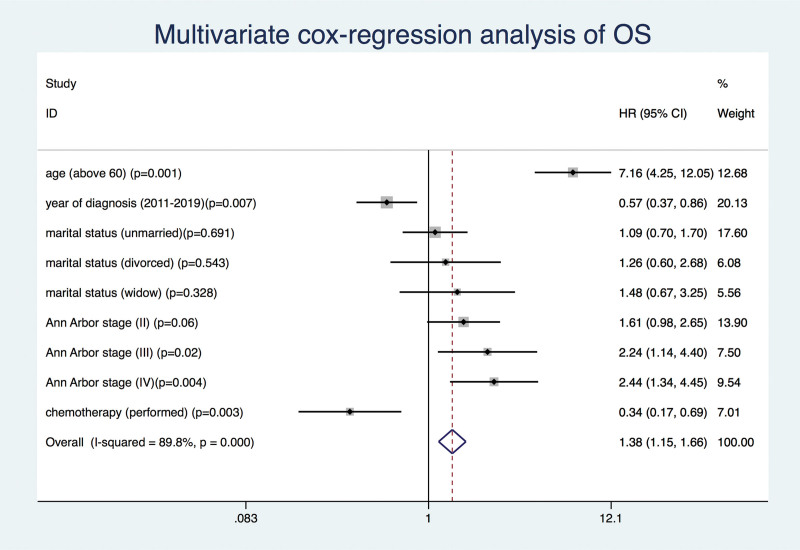
Multivariate cox regression analysis of overall survival in primary mediastinal large B-cell lymphoma patients.

**Figure 8.**
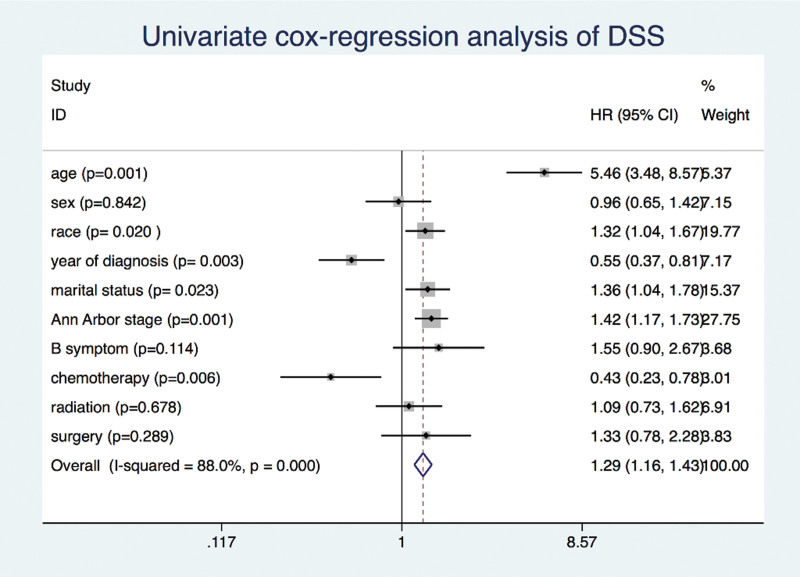
Univariate cox regression analysis of diseases specific survival in primary mediastinal large B-cell lymphoma patients.

**Figure 9.**
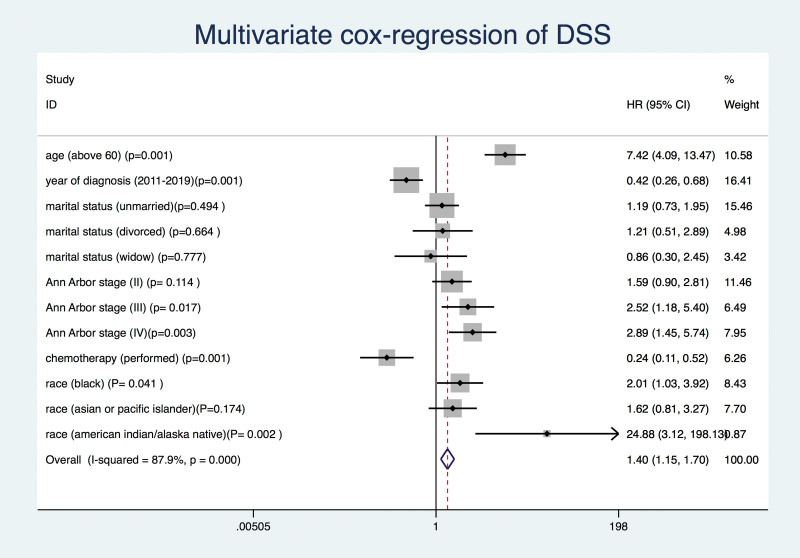
Multivariate cox regression analysis of diseases specific survival in primary mediastinal large B-cell lymphoma patients.

The OS and DSS of all patients with PMBCL were illustrated in Figure 1A and B. A total of 134 patients died by the end of follow-up, of which, 105 deaths were attributed to disease-specific mortality. K-M survival analysis manifested that the median OS and DSS were not reached for the entire patient cohort. One-year, 5-year, and 10-year OS rates were 93.99%, 85.04%, and 81.76%, respectively. Similarly, 1-year, 5-year, and 10-year DSS rates were 95.27%, 87.37%, and 85.98%, respectively (Table 2). Further K-M survival analysis was conducted to assess the impact of various factors on OS and DSS, including age, gender, ethnic group, years of diagnosis, marital condition, Ann Arbor staging, B symptoms presentation, and treatment modalities. The analysis revealed that older patients were significantly associated with inferior OS and DSS (Figs. 2A and 3A). Patients diagnosed between 2011 and 2019 exhibited better OS and DSS rates (Figs. 2B and Fig. 3B). The Ann Arbor staging had a significant influence on both OS and DSS, with worse outcomes observed in advanced stages (Figs. 2C and 3C). Marital status also affected OS and DSS, with widowed patients experiencing poorer survival (Figs. 4A and Fig. 5A). Regarding treatment options, patients underwent chemotherapy had tremendous improvement of OS and DSS compared to those who did not (Figs. 2D and 3D). Conversely, radiation therapy had no impact on OS or DSS (Figs. 4E and 5E), and surgery therapy did not affect either OS or DSS (Figs. 4F and 5F). Other characteristics, such as gender, race, and presence of B symptoms, did not impact on the OS and DSS (Figs. 4B, C, D and 5B, C, D). Univariate Cox regression analysis further uncovered that younger patients, diagnosis between 2010 and 2019, and receipt of chemotherapy were associated with improved overall survival. Conversely, being widowed and having an advanced Ann Arbor stage were associated with lower survival rates (Fig. 6). Multivariate Cox regression analysis confirmed that younger age, diagnosis between 2010 and 2019, receipt of chemotherapy, and early Ann Arbor stage were independently associated with superior OS (Fig. 7). Similarly, univariate Cox regression analysis demonstrated that younger age, white race, diagnosis between 2010 and 2019, and receipt of chemotherapy were associated with better DSS. Conversely, widowhood and advanced Ann Arbor stage were associated with lower DSS rates (Fig. 8). Multivariate Cox regression analysis confirmed that younger age, diagnosis between 2010 and 2019, receipt of chemotherapy, and early Ann Arbor stage were independently associated with improved DSS (Fig. 9). These findings indicate that age, years of diagnosis, Ann Arbor staging, and chemotherapy treatment may all serve as independent predictors of both OS and DSS in patients with PMBCL.

**Table 2:** Survival outcome of PMBCL.

Yr after diagnosis	OS	DSS
1-yr	93.99%	95.27%
2-yr	88.26%	89.69%
3-yr	86.45%	87.98%
4-yr	85.58%	87.37%
5-yr	85.04%	87.37%
6-yr	84.44%	87.37%
7-yr	83.75%	87.12%
8-yr	83.17%	87.12%
9-yr	82.52%	86.78%
10-yr	81.76%	85.98%

DSS = disease-specific survival, OS = overall survival, PMBCL = primary mediastinal diffuse large B cell lymphoma.

### 3.3. Construction and validation of the nomograms

**Figure 10.**
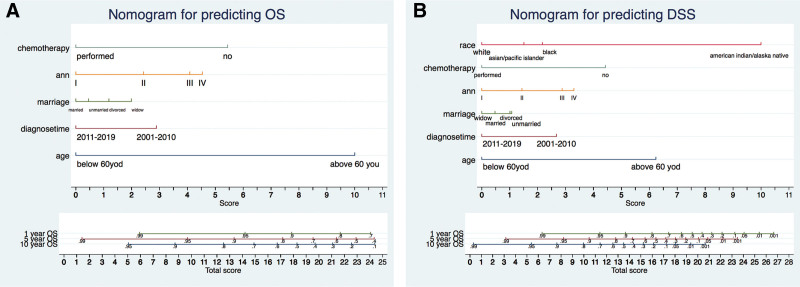
Nomograms for predicting the 1-, 5-, and 10-yr OS (A) and DSS (B) of primary mediastinal large B-cell lymphoma patients. DSS = disease-specific survival, OS = overall survival.

In this study, the multivariate analysis demonstrated that age, years of diagnosis, Ann Arbor staging, and chemotherapy treatment were independently associated with both OS and DSS. Subsequently, all above prognostic factors identified were used to establish a predicting nomogram model. Figure 10A displayed the OS nomogram at 1-year, 5-year, and 10-year intervals, while Figure 10B presented the DSS nomogram at the same time points. By summing the scores assigned to each parameter and projecting the overall scores onto the bottom scale, the likelihood of OS and DSS at 1-year, 5-year, and 10-year intervals could be estimated. Additionally, the concordance probability of the established nomograms was evaluated using Harrell C-index. The C-index for the nomograms predictions of OS and DSS were 0.704 and 0.733, respectively, indicating a high level of accuracy for the constructed nomograms.

## 4. Discussion

This study was undertaken to examine prognostic variables and clinical survival outcomes in PMBCL patients during the rituximab era based on SEER database. The rationale for conducting this study includes the limited availability of large-scale retrospective analyses on PMBCL, the introduction of rituximab chemoimmunotherapy in 2001, and the significant advancements in PMBCL prognosis resulting from the use of novel targeted medications in the first 2 decades of the twenty-first century. A recently published article conducted a population-based study on the incidence and survival of PMBCL, revealing that the age-adjusted annual incidence of PMBCL increased from 0.05 per 1000,000 in 1975 to 2.38 per 1000,000 in 2018, representing a 30-fold increase overall. Furthermore, the study found that there was still an almost 20-fold increase in incidence from 2001 to 2018, when PMBCL was classified as a distinct disease by the World Health Organization in 2001.^[16]^ Our findings on the prevalence of PMBCL from 2000 to 2019 are consistent with earlier publications (data not shown). Our study focuses mainly on predictive variables and clinical survival outcomes in individuals with PMBCL treated with rituximab.

Through the utilization of cox regression analysis, our study has identified several significant independent prognostic factors. Firstly, age has been recognized as a crucial factor in international prognostic index (IPI) scoring systems.^[17]^ Since the SEER database did not provide IPI scores, we investigated the impact of age on the prognosis of PMBCL. By categorizing patients into 2 age groups (below/above 60 years old), we observed that patients under 60 years old exhibited higher survival outcomes in terms of both OS and DSS. Secondly, the advancement of novel drugs in lymphoma treatment, particularly in the first decades of the twenty-first century, has significantly enhanced the prognosis of lymphoma. Consequently, we divided the years of diagnosis into 2 groups (2000–2010/2011–2019) and found that individuals diagnosed between 2011 and 2019 demonstrated improved survival outcomes in relation to both OS and DSS. Lastly, although the Ann Arbor staging system was initially designed for staging patients with Hodgkin disease, it has now become the basis for anatomical staging in non-Hodgkin lymphomas as well.^[18]^ Subsequently, we examined the predictive impact of different Ann Arbor stages on the survival of PMBCL patients and discovered that those in advanced stages (III/IV) had poorer survival outcomes in terms of both OS and DSS.

There is a lack of randomized controlled trial that establish the optimal initial first-line treatment for PMBCL. The available data primarily stems from non-randomized prospective and retrospective studies, which are prone to selection bias. It is important to note that PMBCL is diagnosed based on clinical and pathological factors, which may introduce variability in study comparisons. Nevertheless, considering that PMBCL patients are often of a younger age group, finding a balance between curable aim and potential long lasting toxicities, poses a significant challenge in therapy.^[15]^ In the majority of research investigations, the administration of R-CHOP or R-CHOP-like therapy has demonstrated a 2-year PFS rate of 80% and a 2-year OS rate of 90%. Notably, most studies also combined with local RT, especially when CHOP regimen was utilized, which could potentially lead to long-term complications.^[19–21]^

DA-REPOCH, a treatment regimen was specifically developed for aggressive lymphomas in order to overcome drug resistance. In a phase 2 study conducted by the National Cancer Institute (NCI) involving 51 patients with PMBCL, DA-EPOCHR achieved favorable outcomes (5-year EFS 93%, OS 97%), with only 2 patients requiring RT.^[22]^ Three retrospective studies examined the efficacy of DA-REPOCH versus R-CHOP treatments in PMBCL patients. One study indicated that DA-REPOCH may be more effective, while the other 2 studies found no significant difference in outcomes between the 2 treatment regimens.^[23–25]^ Chemotherapy plays a vital role in the treatment of PMBCL. Our study provides additional evidence supporting the significance of chemotherapy in improving the clinical outcomes and overall survival of PMBCL patients. It has been observed that patients who received chemotherapy had better survival rates. Following the R-CHOP regimen, radiation therapy is commonly administered. However, the impact of RT on the risk of relapse or overall survival, particularly in patients who have achieved complete remission, remains uncertain. Previous retrospective studies have yielded conflicting results, and their findings may be influenced by varying inclusion criteria. Furthermore, the lack of uniform criteria for delivering RT adds to the potential bias in these investigations.^[26,27]^ DA-REPOCH offers a notable benefit by diminishing the necessity for RT. Given the potential for complications, it is advisable for younger individuals to refrain from undergoing RT. There was no observed improvement in survival rates among PMBCL patients who underwent RT in our study. In the conventional approach, the treatment algorithms R/R PMBCL has been similar to that of diffuse large B-cell lymphoma. This involves the administration of salvage therapy, followed by high-dose chemotherapy and autologous stem cell transplant. However, the prognosis for patients with R/R PMBCL is dismal.^[28–30]^ In recent years, novel methodologies including PD1 inhibitors, brentuximab vedotin (BV), and CAR T-cell therapy have demonstrated encouraging outcomes.^[31–34]^

The nomogram, a cutting-edge clinical prediction model, is gaining recognition as an indispensable tool in the medical field. This study has identified several key factors, including age, years of diagnosis, marital status, Ann Arbor stage, and chemotherapy, that independently influence the prognosis of patients with PMBCL, impacting both OS and DSS. Leveraging these factors, we have constructed nomograms capable of accurately predicting survival rates at 1, 5, and 10 years through meticulous multivariate cox regression analysis. To ensure the nomogram reliability and widespread applicability, it must undergo rigorous validation to avoid overfitting. Encouragingly, our investigation has revealed a noticeable improvement in discrimination, as evidenced by the increased C-index of the nomogram. Armed with these nomograms, we can now forecast an individual survival probability at specific time points and develop realistic follow-up plans. Nevertheless, to establish the nomogram credibility, it is imperative to subject it to prospective validation using another independent dataset, thereby enabling a trustworthy evaluation.

Several limitations in this study must be declared. Firstly, it should be noted that our study was conducted retrospectively, which introduces inherent biases that may affect the validity of the findings. Additionally, it is important to acknowledge that there are numerous other factors that can influence survival in PMBCL patients, such as IPI, age-adjusted IPI (aa-IPI), lactate dehydrogenase (LDH) level, bone marrow infiltration, organ involvement, specific front-line induction chemotherapy regimens, time of relapse, salvage therapy for relapsed or refractory patients, and the use of new treatments such as immune checkpoint inhibitors, autologous stem cell transplant, and CART. Unfortunately, the SEER database, which was utilized for this study, does not supply data on these variables, and therefore they were not included in our nomograms. Consequently, it should be cautious in interpreting our results. Despite these limitations, the SEER database remains a valuable resource for studying PMBCL due to its large and diverse population. Our study, despite its drawbacks, has provided important insights into the prognosis and clinical outcomes of PMBCL patients, as well as valuable information on prognostic markers.

## 5. Conclusions

The present study, which focused on the population as a whole, revealed that PMBCL is a relatively uncommon disease. However, its incidence has been consistently rising over the last 20 years. Notably, survival rates have shown improvement in recent times. Rituximab based immunochemotherapy has emerged as an effective treatment option, leading to enhanced OS and DSS outcomes. Furthermore, the nomograms specifically developed for PMBCL have demonstrated robustness and accuracy in forecasting OS and DSS rates at 1, 5, and 10 years. These predictive tools can be valuable for clinicians in accurately estimating prognosis and establishing personalized treatment plans and follow-up protocols.

## Acknowledgments

The authors would like to thank all members of the Department of Hematology, Shanghai Ninth People Hospital Affiliated to Shanghai Jiaotong University School of Medicine, for their support.

## Author contributions

**Data curation:** Haifang Hang, Hui Zhou.

**Formal analysis:** Liyuan Ma.

**Methodology:** Haifang Hang, Hui Zhou.

**Supervision:** Liyuan Ma.

**Writing – original draft:** Liyuan Ma.

**Writing – review & editing:** Liyuan Ma.
